# A new species and a new series of *Elatostema* (Urticaceae) from south-western China

**DOI:** 10.3897/phytokeys.180.65813

**Published:** 2021-08-03

**Authors:** Dan-Hong Yin, Teng-Fei Huang, Zhen Lu, Lin-Dong Duan

**Affiliations:** 1 School of Urban and Rural Construction, Shaoyang University, Shaoyang 422000, Hunan, China Shaoyang University Shaoyang China; 2 Forestry Administration of Shaoyang, Shaoyang 422000, Hunan, China Forestry Administration of Shaoyang Shaoyang China

**Keywords:** *
Elatostema xingyiense
*, series *Xingyiensia*, taxonomy

## Abstract

The new series ElatostemasectionWeddellia series *Xingyiensia* L.D. Duan & D.H. Yin (Urticaceae) is described. In addition, its new species *Elatostemaxingyiense* L.D. Duan & D.H. Yin, endemic to Guizhou Province, is also described and illustrated with photographs. The new series is morphologically similar to series *Melanocarpa* W.T. Wang and series *Sublinearia* W.T. Wang. The new species is most similar to *E.melanocarpum*, *E.sublineare*, *E.obscurinerve*, *E.langicuspe* and *E.youyangense* in morphology, but can be visibly distinguished by a combination of characters, including leaf vein, male inflorescences, female inflorescences and persistent tepals.

## Introduction

The genus *Elatostema* J. R. Forster & G. Forster (1775: 53; Urticaceae) is part of the family Urticaceae and includes about 500 species of sub-shrubs and understorey herbs that grow in the deep shade of forests, gorges, stream sides and caves (Wang 2014; L.F. Fu et al. 2019a). More than 290 species occur in China (Wu et al. 2012) and the greatest species richness occurs on limestone karst in Southeast Asia (Lin et al. 2003; Wang 2014; L.F. Fu et al. 2019b). *Elatostema* is distinguished and characterised from other genera of Urticaceae by its inflorescences of determinate capitula with receptacles and involucres (Z.R. Yang et al. 2011).

We found an unknown species from Guizhou Province, south-western China during our field trips in February 2019 and March 2019. This species is morphologically most similar to *Elatostemamelanocarpum* W. T. Wang, *Elatostemasublineare* W. T. Wang, *Elatostemaobscurinerve* W. T. Wang, *Elatostemalangicuspe* W. T. Wang and *Elatostemayouyangense* W. T. Wang (Wang 1980, 1984, 2013). It differs distinctly from these known species in several morphological features (Table 1) and is described here as a new species.

**Table 1.** Morphological comparison between *E.xingyiense*, *E.melanocarpum*, *E.sublineare*, *E.obscurinerve*, *E.langicuspe* and *E.youyangense*.

	* E. xingyiense *	* E. melanocarpum *	* E. sublineare *	* E. obscurinerve *	* E. langicuspe *	* E. youyangense *
Leaf Veins	Mid-vein impressed, margin revolute	Mid-vein flat, margin flat	Mid-vein flat, margin flat	Mid-vein flat, margin flat	Mid-vein flat, margin flat	Mid-vein flat, margin flat
Male inflorescences	Peduncles 4–20 mm long, dichotomously branched, receptacles cochleariform to oblong, ca. 2–3 mm long, ca. 1–2 mm wide	Unknown	Peduncles 6–10 mm long, single, receptacles inconspicuous	Peduncles 3.5–9.0 mm long, single, receptacles inconspicuous	Peduncles 15–23 mm long, single, receptacles inconspicuous	Peduncles 15–23 mm long, single, receptacles tiny
Female inflorescences	Peduncles 1.0–1.5 mm long, receptacle papilionaceous or elliptic, bipartite, margin indehiscent or lobed, 1.5–6.5 mm long, 1.5–5.4 mm wide, bracts numerous, linear-lanceolate, abaxially puberulent, margin ciliate	Peduncles 1.0–1.5 mm long, receptacle conspicuous or inconspicuous, elliptic, 6 mm long, 3 mm wide, bracts ca. 10, triangular or narrow-triangular, 1.5 mm long, 0.7–3.0 mm wide, ciliolate, abaxially strigulose	Peduncles 1.0–3.5 mm long, receptacle sub-rectangular, 5–7 mm long, indehiscent or bisected. Bracts 50 or more, triangular or narrow-triangular, 0.8–1.2 mm long, densely ciliate.	Peduncles 0.7 mm long, receptacle sub-rectangular, ca. 1.5 mm long and broad. Bracts ca. 17, 2-seriate, broad-ovoid to ovoid, 0.6–1.2 mm long, female flower sessile, ovary ellipsoidal.	Inflorescence sessile. Receptacle sub-orbicular, 1 mm in diam. Bracts 6, narrow-ovoid, 2.5 mm long	Peduncles short and robust, receptacle small. Bracts 8, broad-ovoid, 0.2–0.4 mm long. Bracts missing, ovary subglobose, 0.15 mm long.
Achenes	Sessile, ovoid, ca. 0.35–0.40 mm long, longitudinally 4(–5)-ribbed and tuberculate	Pedicel short, narrow-ovoid, 1.0–1.5 mm long, densely tuberculate, so metimes with numerous short lines.	Pedicel short, elliptic-ovate, 0.6–0.8 mm long, longitudinally 8-ribbed	Unknown	Unknown	Unknown
Tepals	Absent	Ca. 1 mm long	Absent	Absent	Absent	Absent

The genus Elatostema includes four sections, sect. Pellionioides, sect. Weddellia, sect. Elatostema and sect. Androsyce. Based on the designations of sections and series by Wang (2014), the new species is a member of section Weddellia by having minute staminate receptacles. With the presence of a perennial herbaceous habit and penninerved leaves, the new species has traits consistent with ser. Crenata, ser. Nigrialata, ser. Nigribracteata, ser. Sublinearia, ser. Melanocarpa, ser. Stewardiana, ser. Bamaensia and ser. Involucrata. However, the male inflorescences are dichotomously branched, inconsistent with any series listed above. Therefore, a new series is described here.

## Materials and methods

The species specimen was contrasted with the collections at IBK, PE and KUN. A morphological species concept that was developed as part of previous taxonomic research (Wei et al. 2011) was used. All morphological measurements were performed on dried and fresh specimens. Relevant literature was consulted for the identification of specimens (Wang 1980, 1984, 2013). The morphological characteristics of *Elatostemaxingyiense* were determined using a stereomicroscope (Olympus SZX16) integrated camera system (Olympus DP27) and we made the specimen measurements by Olympus cellSens Entry.

## Taxonomy

### Elatostema section Weddellia

Taxon classificationPlantaeRosalesUrticaceae

series Xingyiensia L.D.Duan & D.H.Yin, ser. nov.

ACEE23D9-9651-5C4C-B3AF-80339FD4C9D7

urn:lsid:ipni.org:names:77218851-1

#### Diagnosis.

Mid-vein impressed, margin revolute, pistillate inflorescence peduncle dichotomously branched. ***Typus seriei***: *Elatostemaxingyiense* L.D. Duan & D.H. Yin.

#### Relationship.

The staminate capitula of the species is long peduncelate, as such this new series is closely related to ser. Sublinearia W.T. Wang (Wang 1980). It also has similarity to ser. Melanocarpa W.T. Wang with its achene fawn, ovoid, longitudinally 4(-5)-ribbed and tuberculate (Wang 2013). However, the new series differs from these two series with regards to the following features: leaf mid-vein impressed, the margin revolute (compared to the mid-vein flat and margin flat in ser. Melanocarpa and ser. Sublinearia); pistillate inflorescence peduncle dichotomously branched (compared to the peduncle not dichotomously branched in ser. Melanocarpa and ser. Sublinearia) (Table 1).

### Elatostema xingyiense

Taxon classificationPlantaeRosalesUrticaceae

L.D. Duan & D.H. Yin, sp. nov.

202E2B8F-F660-5CF8-A58F-C5705FE355F8

urn:lsid:ipni.org:names:77218852-1

Fig. 1

#### Type.

China Guizhou: Xingyi City, Maling River Canyon Scenic Area, adarces and walls in the valley floor of middle mountains, 25°09'58.00"N, 104°57'20.07"E, 1110 m alt., 8 February 2019, *Lin-Dong Duan & Zhen Lu*, *6118* (***holotype***: HUSY!, ***isotype*** HNNU!, PE!, HUSY!).

#### Relationship.

This new species is closely related and similar to *Elatostemamelanocarpum* (Wang 2013), *Elatostemasublineare* (Wang 1980), *Elatostemaobscurinerve* (Wang 1980), *Elatostemalangicuspe* (Wang 2013) and *Elatostemayouyangense* (Wang 1984). This new species is visibly distinguished by a combination of characters: leaf mid-vein impressed, leaf margin revolute (mid-vein flat, margin flat in the other five species); pistillate inflorescence peduncle dichotomously branched (not branched in the other five species) (Table 1).

#### Description.

Herbs perennial. Young stems ca. 16–30 cm tall, glabrous, purple, simple, with 3–4 leaves. Leaves sub-sessile, glabrous; blades thin-papery, adaxially green, abaxially purple, obliquely long elliptic, lanceolate-elliptic or ovate-elliptic, 4.0–12.5 cm long, 1.2–3.7 cm wide; apex caudate-acuminate (acumens entire); base sub-orbicular to broad-cuneate at broad side and cuneate at narrow side; margins below mid-leaf entire, above mid-leaf crenate, revolute; venation pinnate, with 3–5 pairs of lateral nerves; cystoliths conspicuous, dense, bacilliform, 0.08–0.16 mm long; stipules subulate, 1.0–1.5 mm long. Mature stems ca. 25–45 cm tall, glabrous, simple or sometimes branched, with female inflorescences near apex with 3–5 leaves. Leaves sub-sessile or shortly petiole, 0.2–4.0 mm long, glabrous; blades papery, obliquely long elliptic, obliquely elliptic to obovate-elliptic, 15–16 cm long, 2.5–6.0 cm wide, apex caudate-acuminate, base broadly cuneate at broad side and cuneate at narrow side; margin below mid-leaf entire, above mid-leaf crenate, margin notably revolute; both surfaces glabrous; venation pinnate, with 4–7 pairs of lateral nerves, adaxially mid-vein impressed, lateral vein impressed near mid-vein, abaxially mid-vein and lateral vein notably ridged, cystoliths conspicuous the same as caulicles. Monoecious, male inflorescence axillary on young stems, female inflorescence axillary on mature stems. Staminate capitula singly axillary, peduncles round, glabrous, 4–20 mm long, apex dichotomously branched, branches 0.5–2.0 mm long, nearly glabrous, capitulum above each secondary peduncle, 3.6–4.8 mm long, 3.6–4.0 mm wide, receptacle cochleariform to oblong, ca. 2–3 mm long, ca. 1–2 mm wide and receptacle 1(–2)-lobed when oblong, glabrous, unilateral bract 3–5 (3 when receptacle is cochleariform, 5 when receptacle is oblong), oval to narrow triangular, 2.0–2.5 mm long, 0.6–2.3 mm wide; apical bract abaxial surface longitudinally 1(–3)-ribbed, ca. 1–2 mm long, abaxial surface nearly glabrous; lateral bract longitudinally 1-ribbed, ca. 1 mm long, abaxial surface nearly glabrous or puberulent, bracteoles few, membranous, semi-hyaline, white, lanceolate; abaxially puberulent with cystolith, margin ciliate, ca. 3 mm long. Staminate flowers peduncles glabrous, 3 mm long; tepals 5, oval, 3 mm long, base connate, glabrous, apex corniculate on 2–3 tepals; stamens 5. Pistillate capitula singly axillary, papilionaceous to quadrangular, 2–8 mm long, 2.0–6.5 mm wide; peduncles 1.0–1.5 mm long, flowers numerous, receptacle papilionaceous or elliptic, bipartite, margin indehiscent or lobed, 1.5–6.5 mm long, 1.5–5.4 mm wide, bracts numerous, linear-lanceolate, black, abaxially puberulent, margin ciliate; bracteoles numerous, linear-lanceolate, black, 1.0–2.6 mm long, abaxially puberulent, margin ciliate. Pistillate flowers sessile, tepals absent, ovary ovoid, stigma penicillate. Achenes brownish, ovoid, ca. 0.35–0.40 mm long, longitudinally 4(–5)-ribbed, tuberculate.

**Figure 1.**
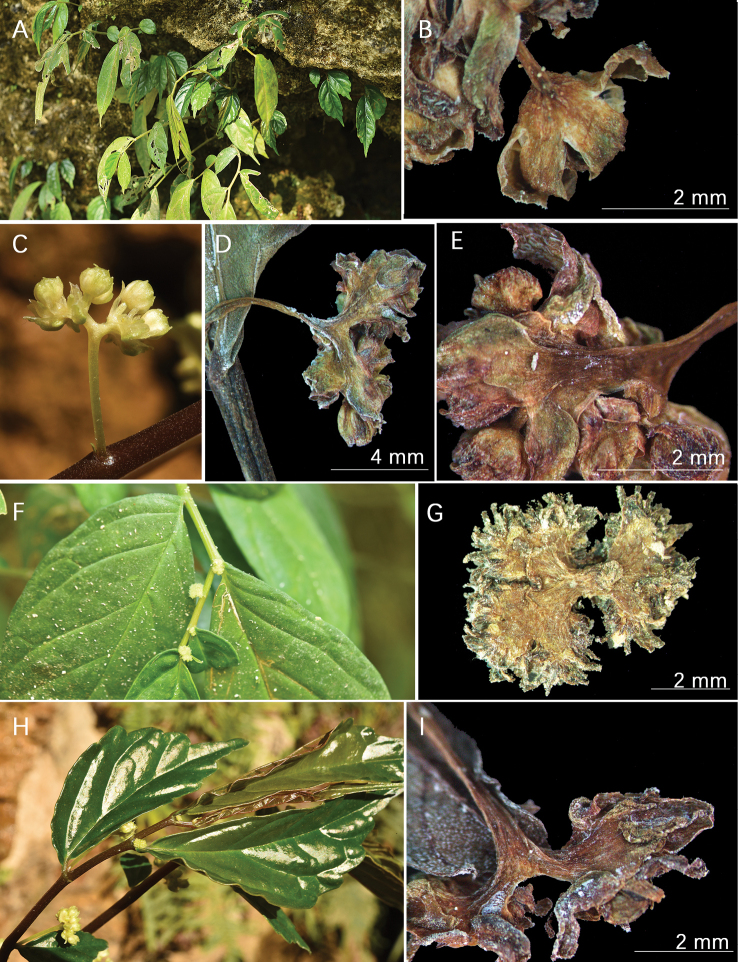
*Elatostemaxingyiense* L.D. Duan & D.H.Yin **A** habit **B** male flower **C** male inflorescence in fresh specimen **D** male inflorescence **E** male inflorescence and secondary peduncle **F** female inflorescence in fresh specimen **G** female inflorescence **H** blades in fresh specimen **I** bract longitudinally 3-ribbed. Photos: Lin-Dong Duan and Dan–Hong Yin.

#### Phenology.

During our field trips, plants were observed in full bloom and without fruits on 10 February 2019, then flowers and fruits on 9 April 2019. The flowering in February to April, fruiting in March to May can be expected.

#### Habitat.

The new species grows on limestone in the valley floor of middle mountains, Maling River Canyon Scenic Area, Xingyi City, Guizhou Province, south-western China.

#### Distribution.

*Elatostemaxingyiense* is only known from one locality in Maling River Canyon Scenic Area, Xingyi City, Guizhou Province, south-western China.

#### Etymology.

The new species was named after its type locality, Xingyi City, Guizhou Province, China.

#### Vernacular name.

兴义楼梯草(Xīng yì lóu tī cǎo) is Chinese Pinyin for *Elatostemaxingyiense*, the first two characters are the place name of Xingyi City, the last three characters are the Chinese name for *Elatostema*.

#### Conservation status.

*Elatostemaxingyiense* is only known from one collection with about 1000 individuals in Maling River Canyon Scenic Area, Xingyi City, Guizhou Province, south-western China (ca. 74 km^2^). This species is under threat because of its fragmented habitat and there is tourism in the type location, Maling River Canyon Scenic Area. It is only in one small area of less than 100 km^2^ and has threats from anthropogenic factors. We suggest that *E.xingyiense* should be considered as “Endangered” (EN) according to the IUCN Red List Categories and Criteria (IUCN 2019).

## Supplementary Material

XML Treatment for Elatostema section Weddellia

XML Treatment for Elatostema xingyiense
